# Design, implementation and operation of a multimodality research imaging informatics repository

**DOI:** 10.1186/2047-2501-3-S1-S6

**Published:** 2015-02-24

**Authors:** Toan D Nguyen, Parnesh Raniga, David G Barnes, Gary F Egan

**Affiliations:** 1 Monash Biomedical Imaging, Monash University, Melbourne, Australia; 2 Monash e-Research Centre, Monash University, Melbourne, Australia; 3 CSIRO, Melbourne, Australia; 4 Faculty of Information Technology, Monash University; VLSCI Life Sciences Computation Centre, Melbourne, Australia; 5 School of Psychology and Psychiatry, Monash University, Melbourne, Australia

## Abstract

**Background:**

Biomedical imaging research increasingly involves acquiring, managing and processing large amounts of distributed imaging data. Integrated systems that combine data, meta-data and workflows are crucial for realising the opportunities presented by advances in imaging facilities.

**Methods:**

This paper describes the design, implementation and operation of a multi-modality research imaging data management system that manages imaging data obtained from biomedical imaging scanners operated at Monash Biomedical Imaging (MBI), Monash University in Melbourne, Australia. In addition to Digital Imaging and Communications in Medicine (DICOM) images, raw data and non-DICOM biomedical data can be archived and distributed by the system. Imaging data are annotated with meta-data according to a study-centric data model and, therefore, scientific users can find, download and process data easily.

**Results:**

The research imaging data management system ensures long-term usability, integrity inter-operability and integration of large imaging data. Research users can securely browse and download stored images and data, and upload processed data via subject-oriented informatics frameworks including the Distributed and Reflective Informatics System (DaRIS), and the Extensible Neuroimaging Archive Toolkit (XNAT).

## Background

Modern clinical and biomedical research is increasingly reliant on imaging across a range of electromagnetic and acoustic wavelengths [1-3]. Contemporary studies now routinely collect images from more than one type of instrumentation - multi-modal studies [4] - and strive to obtain high spatial and/or temporal resolution data. Multi-modal datasets provide complementary information [5] and enable sophisticated, multivariate analysis, while high-resolution datasets provide insight that was not possible only a few years ago. Extremely large multi-modal imaging studies can result in terabyte (TB) size data collections [6], although most research studies generate data in the megabyte (MB) to gigabyte (GB) range per subject.

The data volume per subject is multiplied by the increasing number of subjects per study. Many of today's high profile biomedical imaging studies have hundreds to thousands of participants [7-9]. Furthermore, many of these studies are longitudinal in nature and thus collect imaging data at multiple time points per subject. This multiplier effect results in a large collection of data that must be recorded per subject. Along with the imaging data, non-imaging and meta-data may also collected and should be stored and directly associated with the image data, especially if the data will be mined and/or shared [10].

Clinical informatics systems such as clinical picture archiving and communication systems (PACS) are commonplace [11], but their design, specifically for clinical settings, precludes effective use in a research environment. For example, the majority of PACS store data only in the Digital Imaging and Communications in Medicine (DICOM) format. The DICOM format consists of a binary header of tag/value pairs. The tags (2 bytes) are keys but the descriptions of tags are stored independently in DICOM dictionaries and not in the data itself. The type of the value is contained in the tag/value pair, which enables the accurate reading of the data and meta-data. Binary data is stored as a tag/value pair.

Neuroimaging processing and analysis is however typically conducted using a myriad of proprietary formats such as MINC [12], MRTrix image File (mif) [13] and Freesurfer File Format (mgh) [14]. Recently, the Neuroimaging Informatics Technology Initiative (NIfTI) has provided a reference file format that is starting to become universally accepted and utilised [15]. The reason for the use of non-DICOM format was that traditionally, DICOM data for imaging modalities was stored as a single 2D image per file. For large 3D datasets, this means a lot of repetition of meta-data and slow reading of the data. The newer DICOM 3.0 format has alleviated some of these performance issues but at the expense of simplicity. Moreover the DICOM standards define a set of required meta-data based on the acquisition modality. Many of these required fields do not make sense for processed data and other relevant meta-data would need to be stored as DICOM tags which may not be understandable by all software.

The limitations of a solitary supported image format notwithstanding, it is not possible to keep track of and provide provenance for processed image or sensor data, which is usually in non-DICOM formats such as the NIfTI format. While some proprietary formats include support for meta-data by storing key value pairs, no such ability is present in NIfTI for example. Examples of such meta-data include the diffusion direction table that was used to acquire diffusion magnetic resonance imaging (MRI), control/tag/reference flags for arterial spin labelling images, various reference images and parameters for magnetic resonance (MR) spectroscopy data. Most of this meta-data is encoded as tag/values in the DICOM header but is lost on conversion to other types. Other types of meta-data include descriptions of the type of data (e.g. brain gray matter segmentation) and of the tools and/or pipelines that generated the data. Typically the later is done by utilising common naming conventions. However this can lead to ambiguity if all users and all tools do not implement the convention. Moreover, only a limited amount of information can be stored in this format.

Furthermore the most commonly used DICOM data model is a subject (patient)-centric model. While the DICOM standard allows for a data model that is project-centric, such as the clinical trial information entity, but in practise, PACS usually do not support this feature. The patient or subject centric model in DICOM has been developed with the clinic in mind. Each subject/patient is assumed to be independent of the other with little in common and it is not possible to group subjects together. Moreover the DICOM model does not inherently support the idea of longitudinal studies where the same patient is repeatedly scanned, some time interval apart. The ability to organise and quickly access data based on a project centric data model is essential to research applications which are project centric by nature.

Apart from the need for storing acquired data, research projects require the storage of post-processed data. The raw data is put through various automated and semi-automated algorithms to produce images and well as other data types and statistics. A description of all the processing steps and parameters needs to be stored with the data in order to keep track of how the final data was obtained. This provenance information is crucial in also keeping track of potential changes that may have occurred over different processing runs as well to search for data across projects that maybe similarly acquired and processed.

The need for raw data collection and management, as well the accurate recording of data provenance of processed data, for large biomedical imaging research studies, has resulted in the recent development of software packages, unlike clinical picture archiving and communication systems (PACS), that are designed specifically for research studies [16-19]. Along with the collection and storage of the primary data, these systems have been designed to store processed data as well as provenance information regarding the processing steps [20], although tight integration of the provenance information within the informatics platform is still under active research and development. Currently, in many such systems, provenance information is just another piece of meta-data that is optional. It's formatting and contents are up to the users. With tight integration, the province information would be required, would follow a known format and be ingestible by the system. The difficulty with this is that no universal standard for provenance in medical imaging exists either. Processed data storage and access is a critically important area since the size of processed datasets can be many tens of times larger than the original dataset, and in many cases are expensive to recompute.

While informatics platforms for medical imaging are available, implementing an informatics strategy at a research-focused imaging facility is a challenging task. It depends on integrating acquisition systems (modalities) with good imaging informatics practise realised as a data model-based system, underpinned by archival-grade data storage infrastructure, and complete with functional and practical user interfaces. Most of the informatics platforms are oriented around the Project-Subject-Study-Data (PSSD) model but differ slightly in their implementation details and access methods. In this paper we describe the implementation of the informatics systems and data flows at the Monash Biomedical Imaging (MBI) facility at Monash University. Moreover we describe how we developed a set of tools and standard practises to that have enabled the efficient storage and access of biomedical imaging data.

## Methods

### Requirements

We start by considering a concise listing of the main requirements of an imaging informatics system at Monash Biomedical Imaging. A full requirements specification would be too long for this paper; instead, we focus on the core capabilities needed to support a generic multi-modal, multi-subject, longitudinal study - the core research activity we endeavour to enable and support.

1) imaging data from DICOM capable modalities (e.g. MRI) must be, to a large extent, automatically routed from the point of acquisition to the imaging informatics system;

2) imaging and non-imaging data from non-DICOM capable modalities (e.g. EEG) must be, to a large extent, easily manually uploaded to the imaging informatics system or uploaded using scripts and command line tools;

3) imaging and non-imaging data and meta-data must be stored on secure, reliable, research grade backed-up storage;

4) upon ingest of DICOM-format images, standard meta-data should, to a large extent, be automatically extracted ("harvested") from the DICOM files and recorded in the imaging informatics system;

5) human imaging data must be accessible by standard radiology software for review by the MBI radiologist;

6) imaging and non-imaging data must be organised in a study centric fashion, supporting multi-modal and longitudinal image collections per study subject;

7) an end user tool should exist to aid users in defining the set of meta-data to associate with a study and its subjects, and in defining the data acquisition(s) that comprise the study;

8) all data must be uniquely identifiable without the need for real subject names or identifying information other than date of birth and gender;

9) imaging and non-imaging data and meta-data must be available via a secure web portal to the owner (research leader) and their delegate/s;

10) imaging and non-imaging data must be transferable from within the secure web portal to the accessing workstation ("download") or to Monash University's high performance computing facility MASSIVE ("transfer");

11) users should be able to manually package and upload processed data and record provenance (e.g. link to the source data set/s); and

12) a command-line based tool must be available that enables search of the image informatics system, and upload and download of data collections, for use in batch processing workflows.

### Informatics systems and data model

At MBI we currently have two informatics platforms deployed, namely DaRIS [17] and XNAT [16]. DaRIS is a framework built on the top of Mediaflux (Architecta Pty Ltd, Melbourne, Australia), a commercial media asset management system, and is specifically designed for medical imaging data. Assets in Mediaflux are associated with XML meta-data that can be automatically extracted from data or input manually by users. Mediaflux provides a set of services for data management, such as finding, storing and retrieving assets, archiving and handling large data, and data analysis and transformation. To protect data, Mediaflux implements a strong authorisation model in which role based authorisation is used to access to data and each repository has independent access control. DaRIS builds on these capabilities by imposing a data model and a set of methods.

**Figure 1:**
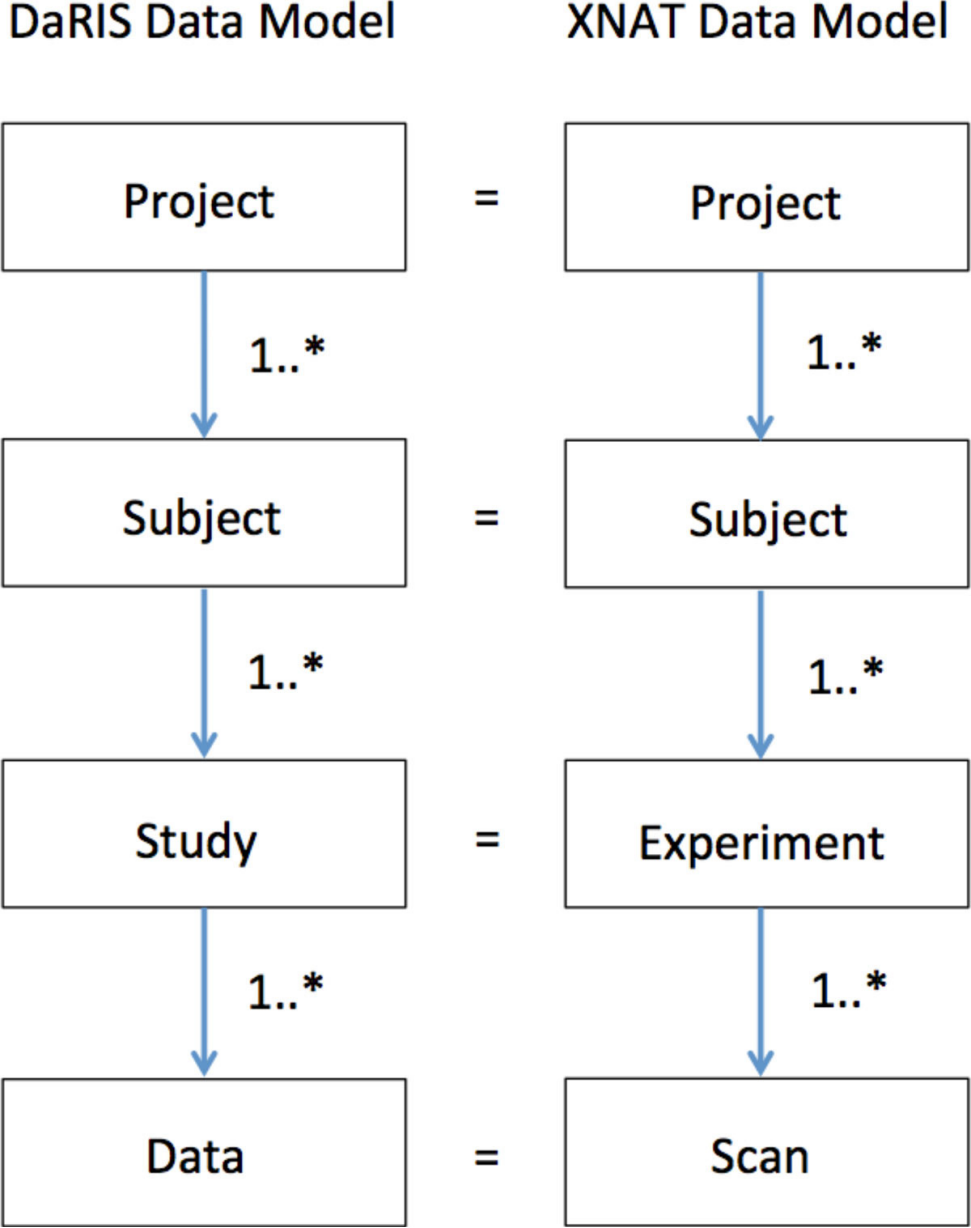
**The data models of DaRIS and XNAT = illustrate the one to one correspondence between two models**. The data model diverges slightly at the Scan/Data level in that XNAT has specific subclasses (Reconstruction and Image Assessment) for post-processed data deriving from Scan whereas this information is contained in the meta-data and methods in DaRIS. Study and experiment are the same concept.

The data model adopted at MBI is the project-subject-study-data (PSSD) model that is used in DaRIS, and although the elements of the XNAT data model are named differently, they can be mapped directly to the PSSD data model (Figure 1). The PSSD data model is a hierarchical data model that is anchored at the project level, unlike the DICOM data model. Each object in the model has an independent *citable identifier *that allows the object to be referenced uniquely in a distributed environment, with the uniqueness property in DaRIS enforced by Mediaflux. A *method *declares what meta-data must and optionally can be entered when a new entity (project, subject, study or data set) is created. New methods can be created using Tool Command Language (TCL) script which Mediaflux natively supports, or Method Builder, which is a Mediaflux web interface plugin developed for us by the Victorian e-Research Strategic Initiative (VeRSI).

XNAT, a free Open Source Software imaging informatics platform, is designed for common management and productivity tasks for imaging and associated data. It has been developed based on a three tiered architecture including a data archive, a user interface and a middleware "engine". The XNAT data model is equivalent to the DaRIS one with project-subject-experiment-data forming the hierarchy. While XNAT does not have an explicit method type like DaRIS, extra data and meta-data can be entered into XNAT. Researchers can work with their data easily using the XNAT web interface to upload data using data entry forms, perform data-type-specific search, view detailed reports and experimental data and access research workflows. Like DaRIS, XNAT has an in-built DICOM server that can be programmed to archive incoming data based on values in specified DICOM tags. XNAT has an HTTP based REST API for querying, retrieving and storing data.

The XNAT and DaRIS data-models diverge slightly at the Scan/Data level in that XNAT has specific subclasses (Reconstruction and Image Assessment) for post-processed data deriving from Scan whereas this information is contained in the meta-data and methods in DaRIS. For primary data, the data models are equivalent. For processed data, XNAT stores the data within reconstructions and image assessments (we only utilise the later) which is a subclass of scan. In DaRIS, no such distinction exists and the associated meta-data at the Data level reflects the difference between primary and processed data.

In order to simply the access to the systems, hide differences between data models and enforce some meta-data entry, we have developed python classes that map onto the PSSD model for interacting with DaRIS and XNAT. These classes hide the lower level interaction from the user and allow them to utilise both in a similar manner. Moreover, the python tools enforce the entry of certain meta-data. For example, provenance information needs to be attached to every processed dataset before it is uploaded. Similarly a description and version of the tools or workflow that produced the dataset needs to be entered.

The python tools are currently being utilised for automated workflows. These workflows are scripts/programs that are designed to download appropriate datasets from projects, perform a task and uploaded processed data back onto the informatics system. An example of this is the Freesurfer recon_all workflow [14] that segments brain MRI images. Another example is the preprocessing of functional MRI data to correct for head-motion and distortion.

### Automatic data flows

**Figure 2:**
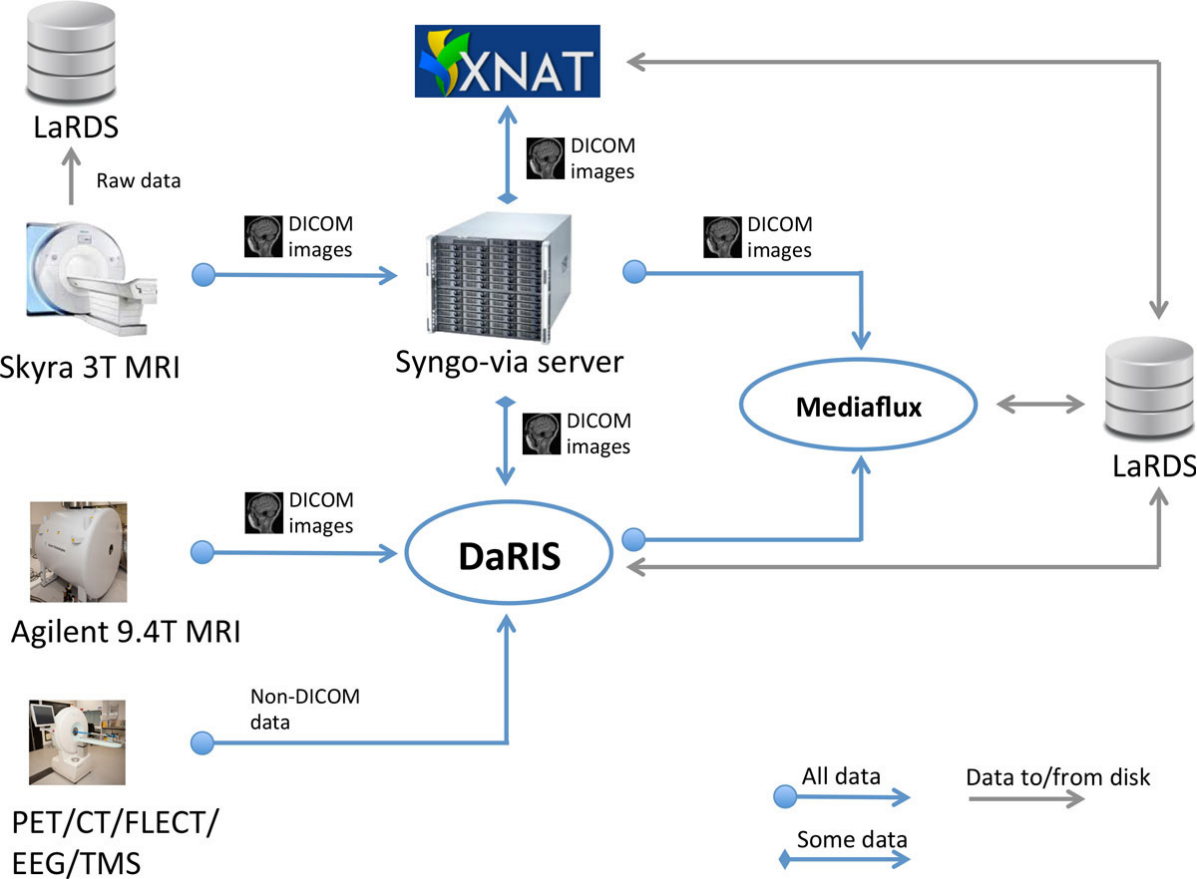
**The acquisition and automated data flows through the current system**. Data from the scanners are pushed directly to DaRIS or to Syngo-via Server and then forwarded to XNAT or DaRIS.

The automatic data flows in the MBI imaging informatics system are shown in Figure 2. Data sources (i.e. scanners) are shown on the left. The Syngo-via server is a clinical PACS system and radiological reporting tool (Siemens Via server, Siemens, Erlangan, Germany). The Monash petascale Large Research Data Storage (LaRDS) system provides the networked, high performance storage and backup system. DaRIS and XNAT are the front-end research informatics platforms described in the previous section that we currently use and support.

DICOM data from human subjects (control or otherwise) MRI scans are sent from the scanner to the Syngo-via server and reviewed by a radiologist for incidental and adverse findings. This data route currently is only used for our 3 Telsa Siemens Skyra MRI scanner (Siemens, Erlangan Germany) but future facilities acquiring imaging data from human volunteers will join into this path. From the Syngo-via server, data is forwarded to one or both research informatics platforms (DaRIS and XNAT currently, but others easily supported) based on the values in specified DICOM tags. All DICOM data arriving at the Syngo-via server are simply forwarded to a secondary DICOM server running on Mediaflux for "last resort" archiving. Whilst DaRIS is our principal (and default) repository for biomedical imaging data and meta-data, XNAT is available where it presents an advantage or preference for the users.

It should be noted that all subjects (human and non-human) are assigned unique identifiers (subject identifier and scan identifier) prior to scanning. Identifying and other associated meta-data for human subjects is stored in a separately maintained, secure, administrative database. For human subjects, no identifying data is stored in the DICOM images apart from gender and date of birth. If a subject must be identified (e.g. for reporting of incidental findings), it is done so via the mapping in the secure administrative database real identities.

A normal clinical PACS operates only on DICOM images via DICOM communication. In a research imaging data management environment raw data (pre-image reconstruction) and non-DICOM image data must also be managed, since pre-clinical imaging scanners do not in general implement full DICOM support. Data from the 9.4T and microPET/CT scanners are sent directly to the associated platforms e.g. DaRIS either automatically via export of DICOMS or semi-automatic uploads of proprietary formats using scripts. Raw data from the 3T Skyra scanner is also sent to a server and then archived to LaRDS on request from projects. This can then be reconstructed and post-processed with different algorithms to those available on the scanner.

For large datasets that are required to be accessed often, digital object identifiers (DOIs) and digital handles are being implemented in DaRIS as long-lived references to the datasets.

### User interaction

**Figure 3:**
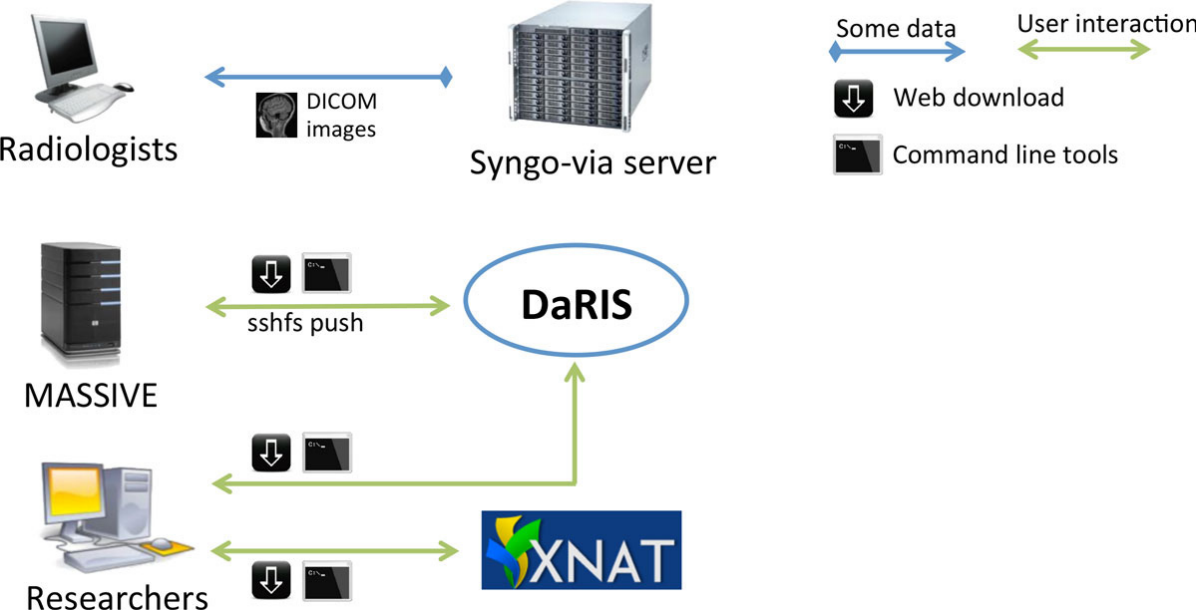
**User interaction**. Illustration of the different ways the user can interact with the informatics system.

Researchers access data stored in DaRIS and XNAT using a web portal, client programs and scripts (Figure 3). Both DaRIS and XNAT implement strong security protocols with role based authenticated access to restrict unintended access. Layered on the permission model of Mediaflux, DaRIS provides four layers of role-based authorisation to protect data objects and services. Each project in DaRIS belongs to users with role-based access that determines their access to assets and services: (i) the nominated project administrator(s) can control access to the project and modify project team/roles, (ii) subject administrator(s) can create and administer subjects, (iii) user(s) can access all research data but not subject identity (where that information has optionally been directly entered by the project or subject administrator - by default subject identity is not stored in DaRIS), and (iv) guest(s) can access meta-data only.

**Figure 4:**
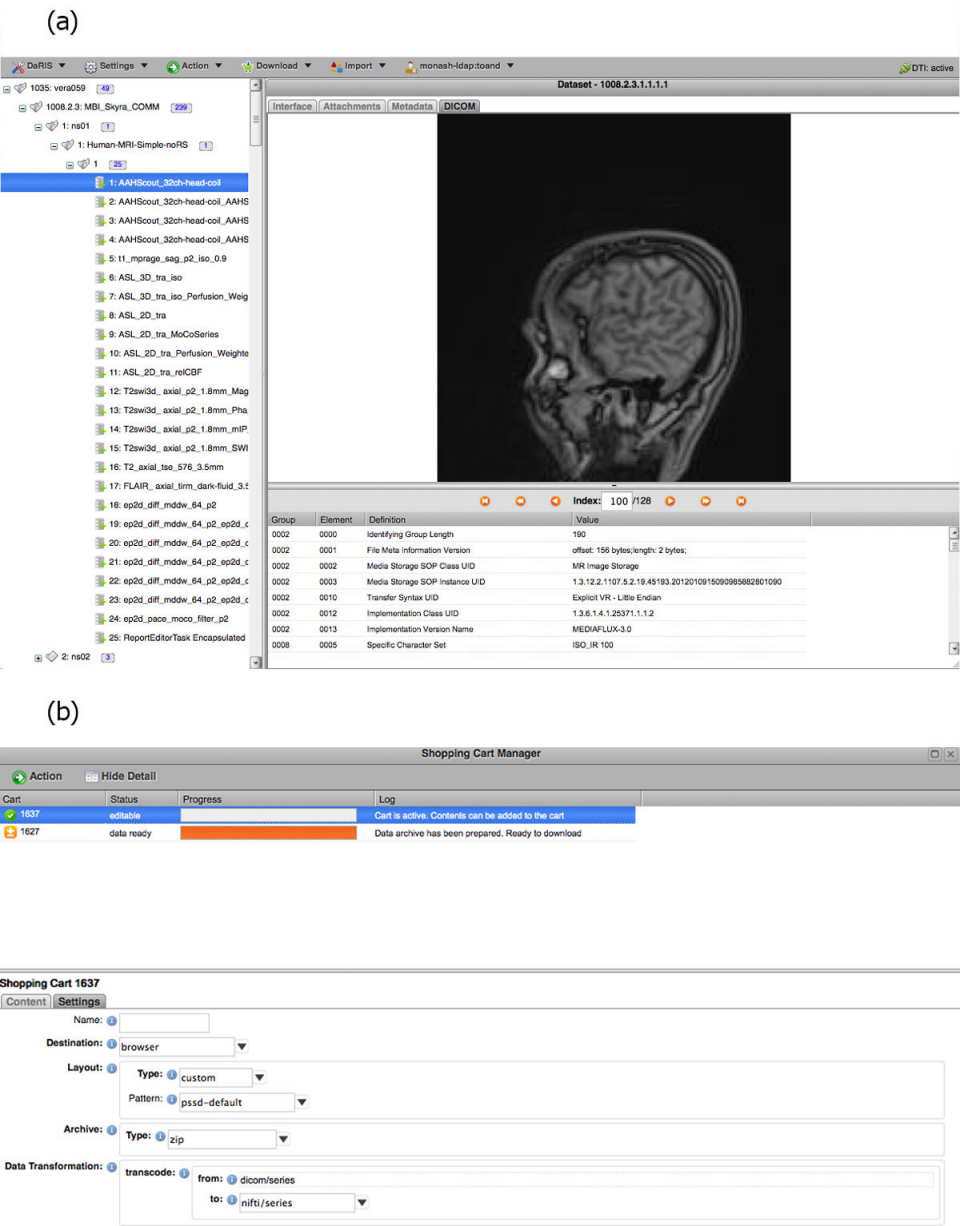
**User interface for DaRIS**. The web based user interface of DaRIS showing the main interface panel in (a) and the "cart" functionality in (b).

DaRIS can operate in a distributed environment and projects can be stored and federated over multiple servers. Since the location of assets associated with data and meta-data is largely transparent to the users and accessible from anywhere through mechanisms including distributed queries, remote access and replication, imaging data stored on DaRIS can be accessed from researchers at different institutions using client programs, command line tools and web portals. As a result, DaRIS provides an efficient way to access data across and within large collaborations. Authenticated users can download data easily using the DaRIS web portal as shown in Figure 4. They can download imaging data of studies, subjects or even an entire project using "shopping carts", and transcoding to popular medical image formats can be applied prior to download. Users can also find and download their data with client scripts which provide a convenient way to process imaging data inside batch scripts or programs developed in their preferred programming languages. For example, users of the Multi-modal Australian ScienceS Imaging and Visualisation Environment (MASSIVE) http://www.massive.org.au high performance computing facility can access and process imaging data that is downloaded from DaRIS.

XNAT can control the access of an individual user down to the single record level by using a hybrid XML relational database structure. When a user retrieves data that are stored in the relational database, XNAT checks whether or not the user is permitted access to the data by using the security protocols defined in XML data model. The XML security protocol is defined by selecting one or more security fields that are assigned one or more allowed values in each user's account. XNAT project administrators can assign access rules for users of the project using administrative tools included in the XNAT web application. XNAT also provides a very flexible HTTP/REST based API for access, control and upload of data. Due to the flexibility of this API and the ability to program XNAT in a language-independent manner, XNAT is a preferred platform for projects that perform significant sequences of automated steps in data management (including on ingest).

Non-DICOM and processed data can be uploaded via the DaRIS and XNAT portals, and using scripts. For DaRIS, post-processed data is added at the same level in the hierarchy as the originating data, but is tagged as being post-processed and referenced to the original dataset. Processed data can originate from more than one data set, as would be the case for a cohort based atlas image in a multi-subject study. In XNAT, post-processed data are tagged as reconstructions or image assessments (subclass of scans). Reconstructions are post-processed raw data or image data that are not derivatives. Image assessments are derivative images or statistics.

## Results

Our system, implemented over the period October 2011 to July 2012 and refined in the intervening time, successfully realises the core capability requirements outlined above. All human imaging projects presently being undertaken at MBI on the Skyra 3T scanner are using the MBI imaging informatics system - and specifically the DaRIS backend - for the management, archive and retrieval of MR images. Many non-human imaging projects, using e.g. the small-bore high-field MR scanner or the small-bore microCT/PET instrument, are also using the system as it provides a simple and reliable image management platform. Additionally, several large projects are being carried out at Monash University using data acquired elsewhere but federated into the Monash University DaRIS system. Large cohort longitudinal studies commenced at MBI will use the imaging informatics system from the outset. Presently (June 2014) there are nearly 100 distinct research projects registered in the system, and 110 users. The total compressed size of ingested data exceeds 1 TB. While this may be considered a relatively small volume, the uncompressed data size is 3-5 times this number. We expect significant growth as large imaging studies get underway and processed data and provenace information are archived together with the raw acquired datasets.

## Discussion

Biomedical imaging studies, especially multi-modal, longitudinal studies of large subject cohorts, generate large collections of data that need to be stored, archived and accessed. Contemporary mid-range MRI based studies can easily accumulate terabytes of data annually. The appropriate use of meta-data, and the recording of provenance for processed data collections, is critical in enabling integrative science, as well as establishing the long term quality and value of the data. The integration of image informatics platforms with the scientific instrumentation, with research quality archival data stores, and with performant processing systems (e.g. compute clusters) is critical in meeting the challenge of extracting new knowledge from biomedical imaging research data.

The system implemented at MBI and described in this article, caters for the needs of a large research imaging centre that generates data from human and non-human imaging experiments. The data is made available to researchers using two informatics platforms, namely DaRIS and XNAT. DaRIS is a project that, while ready for use, is undergoing active development and addition of features. Our close relationship with the DaRIS developers allows us to explore and modify the behaviour of the system to suit, and to provide input on future development directions. Our choice to support XNAT as well is driven by user demand, but effectively positions us to undertake a direct evaluation of the relative strengths, weaknesses, and future opportunities for both systems. In particular, we are very interested in developing interoperability between DaRIS and XNAT to allow flexibility in choice of tool for accessing and manipulating archival image and meta-data. We are also developing a file system based informatics platform based on the python classes. This will give users the ability to either cache their data or to use the python tools and workflows using data from a local filesystem.

Currently, the DaRIS platform is being developed to natively support additional data formats, both standardised and vendor specific formats. This work will enable the automated extraction of relevant meta-data from the supported formats, and the display of image "thumbnails" in the web interface. Another avenue of development is focused on workflows for processing data. Workflows can be programmed in XNAT already but are restricted to run on the XNAT host. Moreover, the pipeline descriptions are programmed using an XML type language specific to XNAT. To alleviate these issues, workflows in DaRIS are currently being developed using the established and well known workflow engines NIMROD [21] and KEPLER [22] and will be designed to distribute computational workload across HPC systems such as MASSIVE and the NeCTAR http://www.nectar.org.au Research Cloud. The automatic provenance tracking already available in Kepler brings a significant advantage presently lacking in XNAT workflows.

Within the context of workflows, we are exploring the choice of "push" versus "pull" processing. The work described above is focussed on push workflows, where (usually implicit) actions within the informatics system initiate processing of data: the data is pushed out to a processing system, the data are processed, and the results ingested. This is appropriate for wholly automated processing of large, rigidly self consistent data sets (i.e. many images that are acquired identically and need to be processed identically), with high throughput. However, for smaller bespoke projects, the pull style of workflow may be more suitable, and in particular enables mostly automated workflows but with manual intervention and inspection. To many users the pull workflow is more natural and controllable. The python tools that have been developed are utilised to develop "pull" type workflows that can be run independently of the informatics system and not tied to any computation platform. For example we have started providing nipype workflows tailored for acquisitions on our scanner for typical neuroimaging tasks. Nipype is workflow/pipeline engine written in python specifically for the medical imaging/neuroimaging community [23]. These workflows are paired with the python tools to download appropriate datasets from projects, perform the task and uploaded processed data back onto the informatics system. An example of this is the Freesurfer recon_all workflow [14] that segments brain MRI images. Another example is the preprocessing of functional MRI data to correct for head-motion and distortion. The advantage of the pull type of workflows is that they are distributed and not confined to the hardware of the informatics system. These allow them to be run from any computer supporting the tools used in the workflow with a cost of data transfer to and from the informatics system.

## Conclusions

A research imaging data management system based on DaRIS and XNAT has been designed and implemented to enable researchers to acquire, manage and analyse large, longitudinal biomedical imaging datasets. The system provides stable long-term storage data and sophisticated support tools for multi-modality biomedical imaging research. Current developments of DaRIS include enhancements to integrate scientific and computational push and pull workflows with the managed data repository. In future work, imaging data will be integrated with the Australian National Data Service (ANDS) registry to make better use of data outputs, and biomedical atlases to provide more quantitative information.

## Abbreviations

API: application programming interface; CT: computer tomography; DaRIS: distributed and reflective informatics system; DICOM: digital imaging and communications in medicine; DOI: digital object identifier; EEG: Electroencephalography; GB: gigabyte; HTTP: hypertext transfer protocol; HPC: high performance computing; LaRDS: large research data storage; MB: megabyte; MBI: Monash Biomedical Imaging; MRI: magnetic resonance imaging; NiFTI: neuroimaging informatics technology initiative; PACS: picture archiving and communication system; PSSD: project-subject-study-data; RAID: redundant array of independent disks; TB: terabyte; TCL: tool command language; VeRSI: Victorian e-research strategic initiative; XML: extensible markup language; XNAT: extensible neuroimaging archive toolkit.

## Competing interests

The authors declare that they have no competing interests.

## Declarations

The authors would like to thank the Monash Biomedical Imaging, Monash University, Melbourne, Australia for financial support.

## Authors' contributions

GFE, DGB and PR developed the background, designed the method and analysed the results. TDN, PR and DGB implemented the methods and provided figures and data. GFE was the leader of this work. All authors read and approved the final manuscript.
